# Management of Agitation During the COVID-19 Pandemic

**DOI:** 10.5811/westjem.2020.5.47789

**Published:** 2020-05-22

**Authors:** Ambrose H. Wong, Lynn P. Roppolo, Bernard P. Chang, Kimberly A. Yonkers, Michael P. Wilson, Seth Powsner, John S. Rozel

**Affiliations:** * Yale School of Medicine, Department of Emergency Medicine, New Haven, Connecticut; † University of Texas Southwestern, Department of Emergency Medicine, Dallas, Texas; ‡ Columbia University, Irving Medical Center, Department of Emergency Medicine, New York, New York; § Yale School of Medicine, Department of Psychiatry, Department of Obstetrics, Gynecology, and Reproductive Sciences, New Haven, Connecticut; ¶ University of Arkansas for Medical Sciences, Department of Emergency Medicine, Department of Psychiatry, Little Rock, Arkansas; || Yale School of Medicine, Department of Psychiatry, Department of Emergency Medicine New Haven, Connecticut; # University of Pittsburgh School of Law and School of Medicine, Department of Psychiatry, Pittsburgh, Pennsylvania

## Abstract

The coronavirus disease 2019 (COVID-19) pandemic caused by the coronavirus SARS-CoV-2 has radically altered delivery of care in emergency settings. Unprecedented hardship due to ongoing fears of exposure and threats to personal safety, along with societal measures enacted to curb disease transmission, have had broad psychosocial impact on patients and healthcare workers alike. These changes can significantly affect diagnosing and managing behavioral emergencies such as agitation in the emergency department. On behalf of the American Association for Emergency Psychiatry, we highlight unique considerations for patients with severe behavioral symptoms and staff members managing symptoms of agitation during COVID-19. Early detection and treatment of agitation, precautions to minimize staff hazards, coordination with security personnel and psychiatric services, and avoidance of coercive strategies that cause respiratory depression will help mitigate heightened risks to safety caused by this outbreak.

## INTRODUCTION

The World Health Organization declared the novel coronavirus disease 2019 (COVID-19) as a pandemic in March 2020, with rising infection rates around the world and within the United States.^1^ This outbreak has radically altered delivery of care in emergency departments (ED), as efforts continue to prevent transmission and combat the disease.^2^ Although attention has appropriately been focused on clinical management and emergency preparedness during COVID-19, this historic event has also had significant consequences for mental health that may be easily overlooked. Unprecedented hardship due to ongoing fears of exposure, threats to personal safety, and limited access to resources have broad psychosocial impact on patients and healthcare workers alike.^3^ These changes can significantly affect how individuals with behavioral symptoms may present and what management strategies are most appropriate during the care of behavioral emergencies.

Agitation is one of the most common behavioral emergencies in the ED, with 1.7 million episodes^4^ annually in emergency settings and a recent estimated overall ED prevalence of 2.6%.^5^ Agitated patients are among the most challenging to evaluate and manage by emergency physicians, as their excessive psychomotor activity can escalate quickly into violent acts and physically aggressive behavior.^6^ Nationwide, 78% of emergency physicians reported being targets of workplace violence in the previous 12 months.^7^ In 2012, the American Association for Emergency Psychiatry (AAEP) published Project BETA (Best practices in Evaluation and Treatment of Agitation), consisting of a landmark series of consensus guidelines to provide effective and safety-minded strategies for agitation management with the best interests of the patient in mind while ensuring the safety of healthcare workers.^4^ The Project BETA guidelines focus on a noncoercive approach to manage these patients with an emphasis on de-escalation, safety and risk assessment, and addressing potentially life-threatening medical concerns.^8^,^9^ Forced medication and physical restraint are reserved as the last resort to control agitation symptoms, given that their use is associated with elevated risk for both patients and staff.^10^

The management principles encapsulated within Project BETA remain applicable in the COVID-19 era, but adaptations are needed in light of the unique circumstances and environmental conditions due to the pandemic. Given the possibility of a projected lengthy timeline before this outbreak abates,^11^ awareness of its effects on the management of agitation is needed now to ensure safety of both patients with behavioral symptoms and frontline healthcare workers caring for them. On behalf of AAEP, we aim to highlight in this work some important unique considerations for the management of agitation in the ED during COVID-19 (Table 1).

## COVID-19 EFFECTS ON PATIENT VISITS AND PRESENTATIONS

### Psychosocial Factors

The COVID-19 pandemic is occurring during a time of unprecedented digital interconnectedness.^12^ Advancements in digital platforms and intense media coverage have amplified the intensity of associated psychological fear, creating a novel “digital pandemic” that significantly exacerbates symptoms of anxiety and stress.^13^ The large-scale public lockdown efforts to implement social distancing has secondarily forced many individuals to stay indoors for prolonged periods of time, increasing the risk of social isolation, tensions within the home, and disruption of positive adaptive behaviors to relieve symptoms of mental illness.^14^ In addition, COVID-19 may directly affect workflow and slow down assessments in the ED, leading to escalation of agitation symptoms for those who require immediate attention.

Hospital visitor restrictions reduce risk of transmission^15^ but also remove vital links of social and family support for individuals during times of crisis. Since asymptomatic carriers can silently transmit the virus,^16^ some patients are fearful that they may unknowingly contract COVID-19 during their time in the ED. Others with symptoms concerning for COVID-19 may escalate their behavior if their expectations for testing or disposition are not met due to limited capacity for EDs to widely test or hospitalize members of the community they serve.^17^ These added pressures can increase the risk of agitation even for visits that may not be associated with a behavioral chief complaint. With reports of recent surges in firearm sales across the US,^18^ extra vigilance is needed regarding potential dangers due to weapons both in the healthcare setting and at home, especially for patients with elevated risk of self-harm or violence.^19^

### Access to Services

Patients presenting with agitation often represent socioeconomically disadvantaged populations with significant health disparities.^20^ Unfortunately, individuals with homelessness, mental illness, and substance use disorders face additional potential problems with screening, quarantine, and symptom treatment during pandemics.^21^ Preliminary data demonstrating associations between mortality and challenges in accessing healthcare resources have already surfaced during COVID-19.^22^ Economic hardship and disruption of outpatient mental health services may limit the ability for these individuals to refill their maintenance medications for psychiatric and/or substance use conditions, causing exacerbation or decompensation of their illnesses. This is compounded by closure of shelters, detoxification units, and other high-density communal settings (eg, drop-in centers and soup kitchens) which may reduce their access to critical social services and increase their likelihood to present to the ED in need. As the support systems and outpatient services deteriorate for these patients, the likelihood that they develop decompensation of their underlying mental illness may increase, leading to ED visits and agitated behaviors during their stay.

### Clinical Presentations

Although it may seem that increased stress and anxiety would inherently increase the volume of behavioral visits during natural disasters and pandemics, experiences from past events have demonstrated that the effects are quite complex and even counterintuitive.^23^ Total mental health-related visits may actually initially decrease as individuals focus on immediate survival and self-protection,^24^ but those who do seek care appear to have more severe symptoms.^25^ For example, inpatient psychiatric admissions fell by 20% for the first 30 days following the devastating earthquake in Christchurch, New Zealand.^26^ New psychiatric presentations following the 2011 Fukushima nuclear plant disaster also decreased, but those admitted had high rates of confusional, manic, and delirious states.^27^ Given the public perceptions of fear and mistrust around the government’s response to the pandemic,^28^ individuals with chronic psychotic disorders may incorporate those perceptions into their delusional content and manifest as themes of contamination, persecution, and conspiracy theories. Particular sensitivity and extra efforts to counteract and redirect these sentiments may be needed as part of the management of agitation.

In addition, there are increasing reports of neuropsychiatric symptoms due to COVID-19. Several case reports have documented encephalopathy and delirium as the presenting syndrome for the disease rather than the more common respiratory or gastrointestinal complaints.^29^,^30^ The Centers for Disease Control and Prevention also found that 6% of hospitalized patients with confirmed COVID-19 had associated symptoms of altered mental status and confusion.^31^ Elderly patients are at the highest risk for morbidity and mortality related to the disease.^32^ Acute agitation in patients with delirium caused by hypoxia, a prominent clinical feature of patients infected with COVID-19, complicates the presentation of dementia and psychiatric illness, particularly in the older population.^33^ Given the elevated rates of clinical and adverse events associated with delirium and the various neuropsychiatric symptoms that may be associated with COVID-19,^34^ emergency physicians need to be mindful of these potential complications when evaluating these patients. A thorough mental status exam^35^ will also help clinicians evaluate the diverse etiologies of any acute behavioral presentation that may be present in this cohort of patients.

## COVID-19 EFFECTS ON CARE DELIVERY

### Individual Staff Factors

COVID-19 has taken its toll on healthcare workers amidst multiple additional stressors imposed upon them.^36^ These include rapid changes in clinical roles and responsibilities, extra workload, disrupted supplies in personal protective equipment (PPE), rationing of resources, and valid fears regarding potential exposure to the disease.^3^ In particular, those on the front lines in the ED may have increased feelings of anxiety, frustration, and resentment due to these added stressors in a dynamic and high-stress clinical environment.^37^ Given that de-escalation requires clinicians to remain calm and compassionate despite displays of aggression or violence, these negative emotions due to COVID-19 can significantly undermine efforts to use patient-centered approaches during management of agitation.^38^

As emergency healthcare workers care for rising volumes of infected patients presenting in extremis, they work at an elevated risk to personal safety from potential occupational exposure to COVID-19.^39^,^40^ This risk increases further during episodes of patient agitation. Clinicians may come into close physical contact with COVID-19 positive patients to de-escalate, provide physical control of disruptive behavior, and perform diagnostic and therapeutic procedures. As a result, professional societies recommend that emergency clinicians continuously wear PPE during their entire shift in the ED. They also note that close contact during procedures or processes, including a physical examination, can generate potentially infectious aerosols and requires a higher level of PPE that includes an N95 respirator.^41^ However, use of PPE may compromise the emergency clinician’s spatial orientation, maneuverability, and awareness of personal safety, which are all vital skills to safely evaluate and manage the agitated patient.^42^,^43^ PPE also adds physical limitations to recognizing facial features and body language, removing key aspects of nonverbal communication that support successful de-escalation and rapport with agitated patients.

### Clinical Resource Limitations

In some geographic areas, EDs are overwhelmed by the volume of COVID-19 infected patients combined with critical shortages of supplies, staffing, and physical space.^44^ Other EDs anecdotally report lower census levels, likely due to a combination of fewer accidental injuries during public lockdown efforts and ED avoidance behaviors by patients fearing exposure to the virus. As a result, staffing models have either decreased or adjusted to focus attention on the surges of COVID-19 cases^45^ and there may be fewer staff available to handle agitated patients in many EDs. In addition, security personnel may have extra responsibilities related to COVID-19 (eg, visitor restrictions, minimizing traffic), impacting the ability for rapid and timely responses to episodes of agitation in the ED. Requirements to ration use of PPE^46^ may further limit the time, attention, and resources normally needed to safely respond to agitation. The increased number of COVID-19 patients with critical care needs has disrupted and limited supplies of sedative medications in the ED.^47^ Ancillary services and psychiatric consultation are also less readily available as they are either furloughed to minimize exposure or deployed to other clinical units with more urgent needs related to the pandemic.^17^ Clinicians need to pre-emptively consider these limitations when managing patients at risk for agitation before behavior escalates and resources are needed rapidly.

## EVALUATION AND MANAGEMENT RECOMMENDATIONS

In a healthcare system that is already taxed with additional stressors on multiple levels, these factors unique to the COVID-19 era discussed above need to be taken into consideration to mitigate escalation to violent behavior and address potential threats to safety associated with agitation. In light of this elevated occupational hazard, extra measures are needed to continually protect the safety of ED personnel and effectively combat an anticipated lengthy battle with this pandemic, regardless of the clinical concerns or level of agitation.^48^ We highlight specific recommendations on the evaluation and management of the agitated patient in the setting of COVID-19.

The medical and psychiatric evaluation should proceed in a manner that minimizes COVID-19 exposure risk while effectively detecting dangerous and reversible causes of agitation. Collateral information should be obtained early to counteract limitations of history taking due to social distancing and PPE requirements. The Joint Statement for Care of Patients with Behavioral Health Emergencies and Suspected or Confirmed COVID-19 supports the use of telehealth for screening,^49^ which may not be applicable in every situation but can significantly reduce exposure. If direct contact is required, donning of appropriate PPE, limiting the amount of time clinicians are less than six feet away from the patient, and minimizing the number of staff members at the bedside will reduce any exposure risk.^3^ The virus has been detected in the saliva of infected patients,^50^,^51^ and precautions must be taken to minimize aerosol and droplet exposure, which may be magnified in those agitated patients who present with pressured speech or spit at ED personnel.^52^ Judicious use and careful consideration of the utility in diagnostic studies are needed to safely evaluate for potentially life-threatening causes of the patient’s agitation. Finally, given known asymptomatic transmission of COVID-19,^53^ there should be a lower threshold to test these patients for the presence of the virus before admission for medical causes of their agitation or transferring them to definitive psychiatric care.

Project BETA strongly encourages early de-escalation, which combines targeted verbal and nonverbal strategies to assist the patient with calming down and reducing aggressive behavior.^8^ In light of COVID-19, extra vigilance and early pre-emptive action are needed to detect and treat any signs of agitation, including use of objective scales to assess the level of agitation and prompt de-escalation by qualified ED personnel. Extra investment in time and effort to develop a therapeutic relationship and establish trust may be needed to overcome additional patient stressors and physical barriers to create rapport. Clinical personnel should communicate early with hospital security if there is any concern about escalation or violent behaviors to allow for lengthier response times and higher potential for escalation, even in milder forms of agitation. Care coordination with psychiatric services is critical in light of limitations to outpatient mental health and social services.

Patients who are delirious and acutely agitated with concomitant COVID-19 infection deserve special attention given elevated patient risks associated with the viral illness. Unfortunately, the ability to implement non-coercive techniques^10^ and reorientation strategies^54^ in treatment of agitation and delirium is compromised by social distancing and isolation measures to minimize COVID-19 spread. Patients who experience persistent and severe agitation or delirium despite de-escalation and attempts to treat underlying causes or precipitants may require physical restraint and use of sedative medication therapy. It is possible that the threshold to use pharmacotherapy may be lower during this pandemic given the elevated risk to both patients and staff caring for them. Low doses of first-generation antipsychotics such as haloperidol or second-generation antipsychotics such as olanzapine and risperidone have been found to be equally effective in patients with delirium, but have differing onset and side-effect profiles.^55^ Extrapyramidal symptoms are most common with haloperidol, and sedation occurs most frequently with olanzapine.^56^ Adverse events associated with restraints and sedatives, including apnea and respiratory depression, will be significantly more dangerous in light of discordance between clinical and imaging evidence for degree of pulmonary involvement, rapid deterioration in the clinical course, and profound hypoxia associated with COVID-19.^57^ If these pharmacologic measures are required, the patient should be closely monitored with frequent vital signs and continuous cardiac, pulse oximetry, and capnometry monitoring.

## CONCLUSION

The COVID-19 pandemic has created unique stressors that may contribute to agitation symptoms. It has also increased personal risks for healthcare staff working in the ED, while adding new limitations to appropriately and effectively manage agitation due to measures needed to combat viral transmission. Extra measures for early detection, treatment of underlying causes for agitation, precautions to minimize staff hazards, coordination with security and psychiatric services, and avoidance of coercive strategies that cause respiratory depression will help mitigate heightened risks to safety caused by this outbreak.

## Figures and Tables

**Table 1:** Summary of COVID-19 effects.

Effects on visits and presentations	
Psychosocial factors	Increase in stress/anxiety symptoms exacerbated by digital media Public lockdown increases tensions between individuals in constant close proximity at home & disrupts healthy coping mechanisms Stress/anxiety due to banning of visitors and fear of COVID-19 exposure when in the hospital Extra vigilance regarding potential weapons on patients given increase in firearm purchases
Access to services	Patients are likely socioeconomically disadvantaged and suffer more during COVID-19 Limited access to their prescribed psychiatric/substance use disorder medications Challenges accessing social services, detox centers, homeless shelters
Clinical presentations	Individuals with milder symptoms may refrain from coming to ED Patients may be in more severe forms of agitation and delirium Possible COVID-19 encephalopathy and delirium syndromes Fears regarding the pandemic may incorporate/feed into delusional content

Effects on care delivery	

Individual staff factors	Staff stress/anxiety levels are high during COVID-19 Risk to personal safety is elevated from viral transmission and may be compounded during episodes of physical violence Maneuvering, spatial orientation, awareness of safety, establishing rapport, attempting de-escalation can be limited by being in PPE
Clinical resource limitations	Ancillary services (chaplain, social work) and psychiatric consultation (deployed elsewhere) may be limited during COVID-19 Medications may be on limited supply due to increased need in ICUs (eg, sedatives) Lower staffing and slower responses from security personnel due to lower clinical volumes and need to conserve PPE

Evaluation and management recommendations to reduce/address agitation	

Evaluation	Obtain collateral information early Perform components of the physical exam from a distance if accurate and feasible Don appropriate PPE and minimize number of staff in direct contact with patient Consider judicious use of diagnostic studies Lower threshold for COVID-19 testing before definitive psychiatric evaluation
Management	Pre-emptive action and extra vigilance to detect and treat early signs of agitation and escalating behavior Prompt and careful coordination with security personnel and psychiatric services Budget extra time and effort for de-escalation and non-coercive strategies Treat underlying cause or precipitants of delirium Caution with sedatives (especially benzodiazepines) and physical restraints for COVID-19+ patients

*ED*, emergency department; *PPE*, personal protective equipment; *ICU*, intensive care unit.
